# Prenatal Exposure to Respiratory Syncytial Virus Alters Postnatal Immunity and Airway Smooth Muscle Contractility during Early-Life Reinfections

**DOI:** 10.1371/journal.pone.0168786

**Published:** 2017-02-08

**Authors:** Paul M. Brown, Terri J. Harford, Vandana Agrawal, Belinda Yen-Lieberman, Fariba Rezaee, Giovanni Piedimonte

**Affiliations:** 1 Center for Pediatric Research, Lerner Research Institute, Cleveland Clinic Foundation, Cleveland, Ohio, United States of America; 2 Pediatric Institute and Children’s Hospital, Cleveland Clinic Foundation, Cleveland, Ohio, United States of America; Imperial College London, UNITED KINGDOM

## Abstract

Maternal viral infections can have pathological effects on the developing fetus which last long after birth. Recently, maternal-fetal transmission of respiratory syncytial virus (RSV) was shown to cause postnatal airway hyperreactivity (AHR) during primary early-life reinfection; however, the influence of prenatal exposure to RSV on offspring airway immunity and smooth muscle contractility during recurrent postnatal reinfections remains unknown. Therefore, we sought to determine whether maternal RSV infection impairs specific aspects of cell-mediated offspring immunity during early-life reinfections and the mechanisms leading to AHR. Red fluorescent protein-expressing recombinant RSV (rrRSV) was inoculated into pregnant rat dams at midterm, followed by primary and secondary postnatal rrRSV inoculations of their offspring at early-life time points. Pups and weanlings were tested for specific lower airway leukocyte populations by flow cytometry; serum cytokine/chemokine concentrations by multiplex ELISA and neurotrophins concentrations by standard ELISA; and *ex vivo* lower airway smooth muscle (ASM) contraction by physiological tissue bath. Pups born to RSV-infected mothers displayed elevated total CD3^+^ T cells largely lacking CD4^+^ and CD8^+^ surface expression after both primary and secondary postnatal rrRSV infection. Cytokine/chemokine analyses revealed reduced IFN-γ, IL-2, IL-12, IL-17A, IL-18, and TNF-α, as well as elevated nerve growth factor (NGF) expression. Prenatal exposure to RSV also increased ASM reactivity and contractility during early-life rrRSV infection compared to non-exposed controls. We conclude that maternal RSV infection can predispose offspring to postnatal lower airways dysfunction by altering immunity development, NGF signaling, and ASM contraction during early-life RSV reinfections.

## Introduction

Respiratory syncytial virus (RSV) is the leading cause of lower respiratory tract infection (LRTI) in children under 5 years of age worldwide and is hallmarked by potentially life-threatening bronchiolitis and pneumonia [1, 2]. Furthermore, strong epidemiologic evidence associates infant RSV LRTI with increased risk of wheezing episodes and asthma later in life [3–8]. Despite this relationship, the exact mechanisms by which early life RSV LRTI predispose to chronic airway dysfunction remain poorly understood. Host immune responses and lower airway inflammation produced during RSV LRTI is clearly of great importance in clearing RSV infection and influence disease severity outcomes [9, 10]. In particular, cytotoxic T lymphocytes are central in the control of active infection and viral clearance, which explains why immunocompromised individuals with deficient cell-mediated immunity experience more severe and prolonged RSV disease and shed the virus much longer [1, 11].

Chronic airway dysfunction developing after early-life RSV LRTI results also from virus-driven modulation of local nerve growth factor (NGF) expression leading to increased neurotransmitters release and neuronal hyperreactivity [12–14]. Accordingly, increased NGF expression and cholinergic innervation were demonstrated within the lower airways of fetal rats exposed to RSV *in utero* [15], without significant change of the other key neurotrophin brain-derived neurotrophic factor (BDNF) [16]. The same study demonstrated the presence of a transplacental route of RSV transmission, the ability of this virus to infect fetal lower airway epithelium, and non-specific airway hyperreactivity (AHR) during postnatal RSV reinfection [15].

Among several aspects requiring additional investigation, the influence of maternal RSV infection on postnatal offspring immunity, neurotrophins expression, and mechanism of airway smooth muscle contractility during postnatal RSV LRTI remains largely unknown. Recently, vertical transmission of viral antigens was reported to impact postnatal immunity whereby macaques exposed to viral epitopes *in utero* displayed altered immunity after postnatal virus challenge [17]. Regarding RSV, the concept of maternal-to-fetal transmission during pregnancy is not unrealistic as evidenced by the documentation of multiple sites of extrapulmonary RSV infection [18–25]. Yet, the idea that a pregnant woman infected with RSV could potentially transmit the virus to her unborn child was unheard of until only recently and raises genuine concerns for potential implications in the pathogenesis of chronic airway diseases. Indeed a very recent document from the Advisory Committee on Immunization Practices of the Center for Disease Control and Prevention (CDC) has recommended the immunization of pregnant women to prevent maternal to infant transmission of the infection [26].

Our previous discovery of vertical RSV transmission led us to investigate whether *in utero* exposure to RSV influences specific aspects of cell-mediated host immunity and airway smooth muscle contractility during postnatal reinfections. We feel the results of this study demonstrate that maternal RSV infection conveys lasting effects on postnatal offspring immunity, which coincide with elevated NGF expression and airway smooth muscle contractility during recurrent early-life RSV LRTI.

## Results

### Maternal rrRSV infection: experimental approach

**Fig 1:**
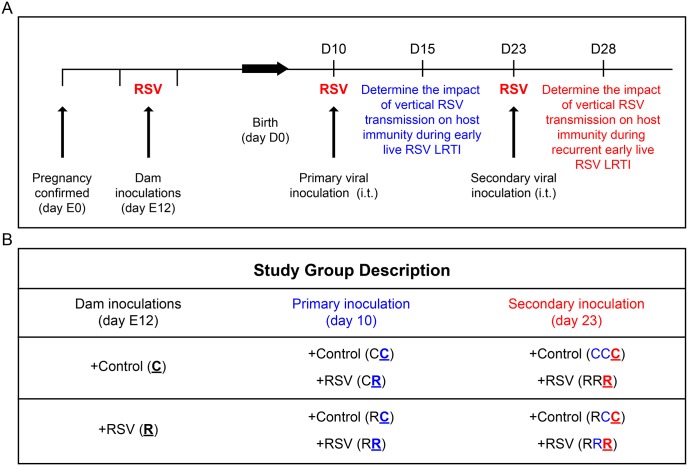
Study design. (A) Timeline detailing prenatal and postnatal inoculation strategy. Female rats were timed for pregnancy and inoculated with rrRSV or virus-free medium at midterm on the 12^th^ day of gestation (E12). Offspring were then inoculated with rrRSV or virus-free medium on either postnatal day 10 (D10) only, or sequentially at D10 and D23. Terminal data collection was performed 5 days post-inoculation, at D15 or D28 respectively. (B) Summary of study groups based on: i.) Prenatal rrRSV exposure status (C vs. R) from dam inoculation on E12; ii.) Primary postnatal rrRSV inoculation at D10; and iii.) Primary rrRSV inoculation at D10 combined with secondary rrRSV inoculation at D23.

To determine if maternal infection with rrRSV impacts the development of postnatal offspring immunity during early-life rrRSV infections, we bred Fischer-344 rats and confirmed pregnancy through vaginal swabbing to time gestation. Dams were inoculated intratracheally at mid-gestation (day E12) using recombinant RSV strain A2 expressing red fluorescent protein (rrRSV; 4 × 10^6^ PFU; Fig 1A) or an equal volume of virus-free control medium. Term offspring were then inoculated with control medium or rrRSV (4 × 10^5^ PFU) at postnatal day 10 (D10) only, or sequentially at D10 and D23 (5 × 10^5^ PFU; Fig 1B).

### Maternal rrRSV infection modifies lower airway T cell pattern in offspring with postnatal rrRSV LRTI

**Fig 2:**
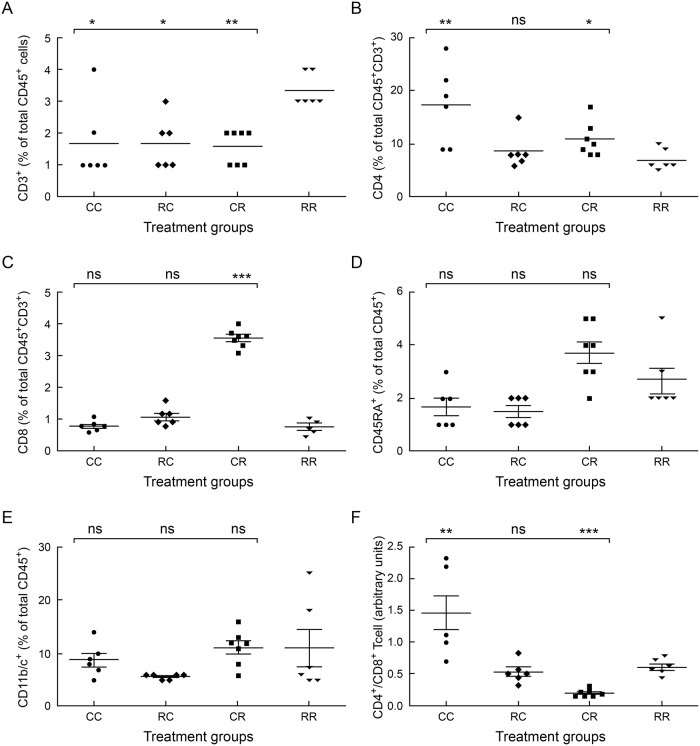
Pups from rrRSV-infected mothers exhibit skewed lower airway T cell populations during primary early-life rrRSV LRTI. Cells purified from BAL of 15-day old rat pups at the peak of rrRSV LRTI were profiled using flow cytometry to quantify populations of: (A) CD3^+^, (B) CD4^+^, and (C) CD8^+^ T cells; as well as (D) B cells and (E) monocyte lineage cells. (F) The mean CD4^+^/CD8^+^ T cell ratio is shown for each experimental group. Data for 3 experiments were combined and expressed as the mean ± SEM of ≥6 rats per group. Data are expressed as percentage of total CD45^+^ cells or total CD45^+^/CD3^+^ cells. Two-tailed student’s *t* test: *P<0.05, **P<0.01, ***P<0.001 compared to the RR group.

To determine if maternal rrRSV infection alters offspring lower airway immunity during early-life rrRSV LRTI, we collected bronchoalveolar lavage (BAL) from pups during peak infection at D15 and quantified cell populations using flow cytometry. We observed elevated CD3^+^ T cells in pups from rrRSV-infected mothers during postnatal rrRSV LRTI (RR) compared to age-matched controls (CR; 3.33% ± 0.21% vs. 1.57% ± 0.2%; *P*<0.0001; Fig 2A). Further characterization of CD3^+^ T cells demonstrated reduced CD4^+^ T cell counts in the RR group during primary early-life rrRSV infection compared to RSV-naïve controls (CR; 7% ± 0.82% vs. 10.86% ± 1.22%; *P*˂0.05; Fig 2B). Additionally, reduced CD8^+^ T cells were observed in RR vs. CR pups as well (11.83% ± 1.3% vs. 56.71% ± 1.8%; *P*<0.0001; Fig 2C).

In contrast to the changes in T cell subpopulations, we found only smaller, non-significant differences in B cell (Fig 2D) and monocyte lineage (Fig 2E) cell counts during primary postnatal rrRSV infection. CD4^+^/CD8^+^ T cell ratios, confirmed the extent of CD8^+^ T cell suppression in RR pups compared to the CR pups (0.6 ± 0.05 vs. 0.19 ± 0.02 arbitrary units; *P*<0.0001; Fig 2F). Furthermore, lower airway CD8^+^ T cell counts were equivalent in RR and RC pups lacking postnatal rrRSV exposure, suggesting altered immune response to rrRSV in RR pups as a consequence of prenatal exposure.

### Elevated CD3^+^CD4^neg^CD8^neg^ T cells persist during recurrent postnatal rrRSV LRTI

**Fig 3:**
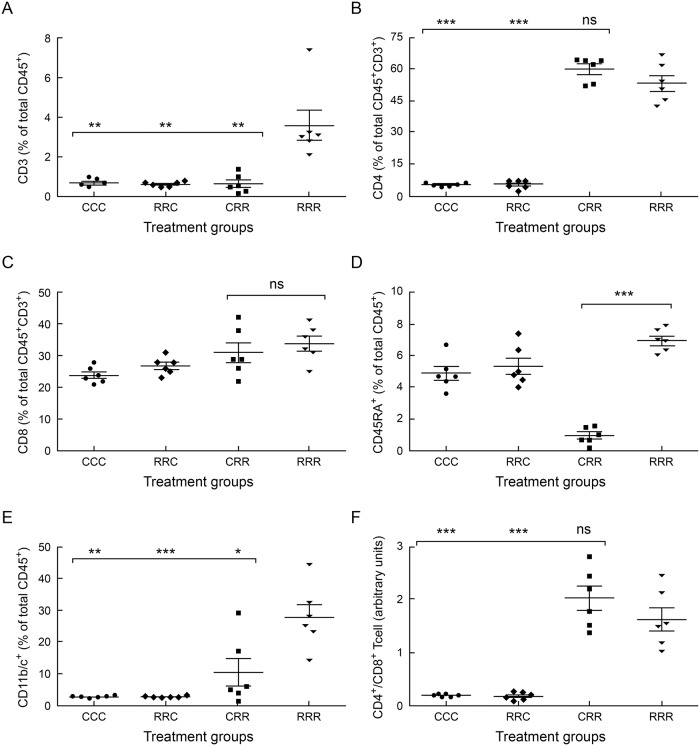
Elevated CD3^+^CD4^neg^CD8^neg^ T cells persist during recurrent early-life rrRSV LRTI. Cells isolated from BAL of 28-day old weanling rats at the peak of rrRSV LRTI were profiled using flow cytometry to quantify populations of: (A) CD3^+^, (B) CD4^+^, and (C) CD8^+^ T cells, as well as (D) B cells and (E) monocyte lineage cells. (F) The mean CD4^+^/CD8^+^ T cell ratio is shown for each treatment group. Data for 3 experiments were combined and expressed as the mean ± SEM of 6 rats per group. Data are expressed as percentage of total CD45^+^ cells or total CD45^+^/CD3^+^ cells. Two-tailed student’s *t* test: *P<0.05, **P<0.01, ***P<0.001 compared to the RRR group.

Prior infection with RSV does not produce lasting immunity and demonstrates the virus’ ability to evade host immune mechanisms. Therefore, we sought to assess whether recurrent postnatal rrRSV infections impact lower airway immunity. We again observed markedly elevated CD3^+^ T cells (*P*<0.01) in weanling rats from rrRSV-infected mothers during sequential postnatal rrRSV LRTI (RRR) compared to all other groups (Fig 3A). Increased CD3^+^ T cell counts observed in RRR weanlings seem the result of maternal rrRSV infection, as CRR weanlings displayed comparatively less CD3^+^ T cells (3.7% ± 0.94% vs. 0.67% ± 0.16%; *P*<0.0001). CD3^+^ T cell counts observed in CRR weanlings after repeated postnatal rrRSV inoculation were equivalent to CD3^+^ T cell counts seen in the non-infected CCC weanlings. Unexpectedly, CD4^+^ and CD8^+^ T cell levels were similar in CRR and RRR weanlings (Fig 3B and 3C). This finding suggests that maternal rrRSV infection exerts a lasting influence on lower airway T cells counts in offspring during recurrent postnatal rrRSV LRTI, but this is not reflective of overall CD4^+^ and CD8^+^ subsets that instead tend to normalize after the primary infection.

Opposite to our findings in D15 pups, lower airway B cells were significantly reduced in CRR compared to RRR weanlings during a secondary postnatal rrRSV infection (0.95% ± 0.22% vs. 6.96% ± 0.36%; *P*<0.0001; Fig 3D), and total lower airway B cells in RRR weanlings were not significantly different from those of CCC and RCC weanlings. Similarly, lower airway monocyte lineage cells were significantly elevated in RRR compared to CRR weanlings at D28 (26.8% ± 4.89% vs. 10.42% ± 4.31%; *P*˂0.05; Fig 3E). Also, unlike what we observed in D15 pups, the CD4^+^/CD8^+^ T cells ratio was similar between RRR and CRR weanlings at D28 (1.64 ± 0.22 vs. 2.03 ± 0.23 arbitrary units; *P* = 0.246; Fig 3F). Collectively, these data suggest prenatal exposure to RSV fails to maintain lasting suppression of lower airway CD8^+^ T cell levels after repetitive postnatal RSV infections. However, CD8^+^ T cell activation status in RRR and CRR weanlings was not evaluated and remains important to characterize in future studies. The effect of maternal infection on lower airway cell counts in offspring during early-life rrRSV infections at D15 (RR vs. CR) and D28 (RRR vs. CRR) are summarized in Table 1.

**Table 1:** Maternal rrRSV infection is followed by altered lower airway immune cell profiles in offspring during postnatal rrRSV LRTI.

Immune cells	RR vs. CR	P value	RRR vs. CRR	P value
**CD3+**	↑↑↑	<0.0001	↑↑	0.0071
**CD4+**	↓	0.0282	↓	ns
**CD8+**	↓↓↓	<0.0001	=	ns
**B cells**	↓	ns	↑↑↑	<0.0001
**Monocytes**	=	ns	↑	0.0327

Comparison of specific lower airway cell populations observed in BAL during primary (RR vs. CR, n ≥ 6 pups per group) vs. recurrent (RRR vs. CRR, n = 6 weanlings per group) postnatal rrRSV LRTI. Data shown are compiled from Figs 2 and 3 and represent 3 independent experiments for each group. Arrows indicate an increase (↑) or decrease (↓) in cell counts. Number of arrows reflects the level of statistical significance calculated using two-tailed student’s *t* test, where *P*<0.05 = 1 arrow, *P*<0.01 = 2 arrows, and *P*<0.001 = 3 arrows.

### Maternal rrRSV infection alters cytokine expression in offspring with early-life rrRSV LRTI

**Fig 4:**
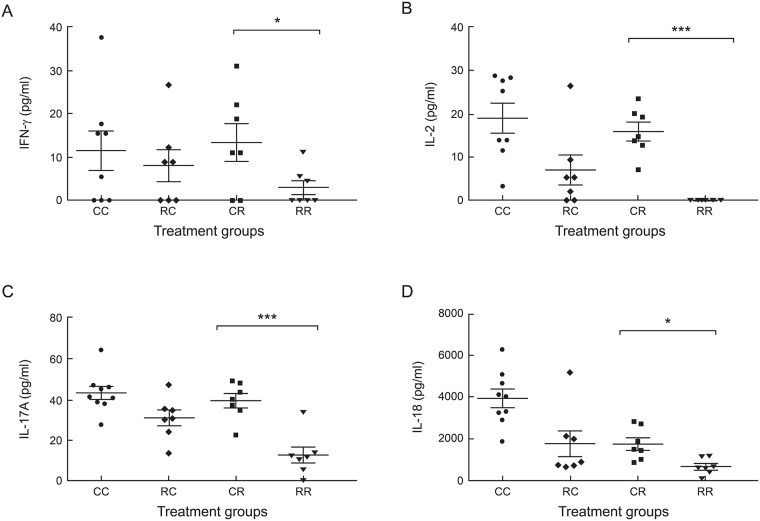
Maternal rrRSV infection alters cytokine expression in offspring with early-life rrRSV LRTI. Serum harvested from 15-day old rat pups at the peak of rrRSV LRTI was analyzed using multiplex cytokine/chemokine ELISA. Data show that maternal rrRSV infection (RR) significantly reduces: (A) IFN-γ, (B) IL-2, (C) IL-17A, and (D) IL-18 in offspring with primary early-life rrRSV LRTI compared to RSV-naïve controls (CR). Data for 3 experiments were combined and expressed as the mean ± SEM of ≥7 rats per group. Two-tailed student’s *t* test: *P<0.05, ***P<0.001 compared to the RR group.

Serum samples were harvested from D15 pups during the peak of rrRSV infection and multiplex ELISA was used to measure the levels of 27 cytokines and chemokines. Compared to controls (CR), pups from rrRSV-infected mothers with early-life rrRSV reinfection (RR) displayed markedly reduced levels of IFN-γ (2.99 ± 1.61 pg/ml vs. 13.41 ± 4.31 pg/ml, *P*˂0.05; Fig 4A), IL-2 (0.0 ± 0.0 pg/ml vs. 15.98 ± 2.07 pg/ml, *P*<0.0001; Fig 4B), IL-17A (12.75 ± 3.94 pg/ml vs. 39.58 ± 3.41 pg/ml, *P*<0.001; Fig 4C), and IL-18 (653.4 ± 144.7 pg/ml vs. 1,745 ± 611.2 pg/ml, *P*<0.01; Fig 4D). Remarkably, while IL-2 was amply detected in all other D15 groups, it was undetectable in all RR pups tested across multiple independent experiments and multiplex ELISA.

Serum IL-2 was also reduced in RC compared CC pups (7.01 ± 3.51 pg/ml vs. 24.71 ± 6.39 pg/ml; *P*˂0.05), suggesting maternal rrRSV infection conveys lasting modifications of early-life immune development, while the profound IL-2 suppression in RR pups indicated that competent activation of effector T cells in response to rrRSV antigens is not effective at generating proficient Th1 immunity as it has been reported in human studies [27, 28]. Similarly, IL-18 data support the hypothesis that deficient IFN-γ production in RR pups was due to reduced macrophage activation during acute rrRSV LRTI, as alveolar macrophages have been shown to express IFN-γ in response to IL-18 signaling [29] and we found no significant difference in lower airway monocyte lineage cells between CR and RR pups at D15.

### Maternal rrRSV infection conveys lasting suppression of cytokines expression in offspring

**Fig 5:**
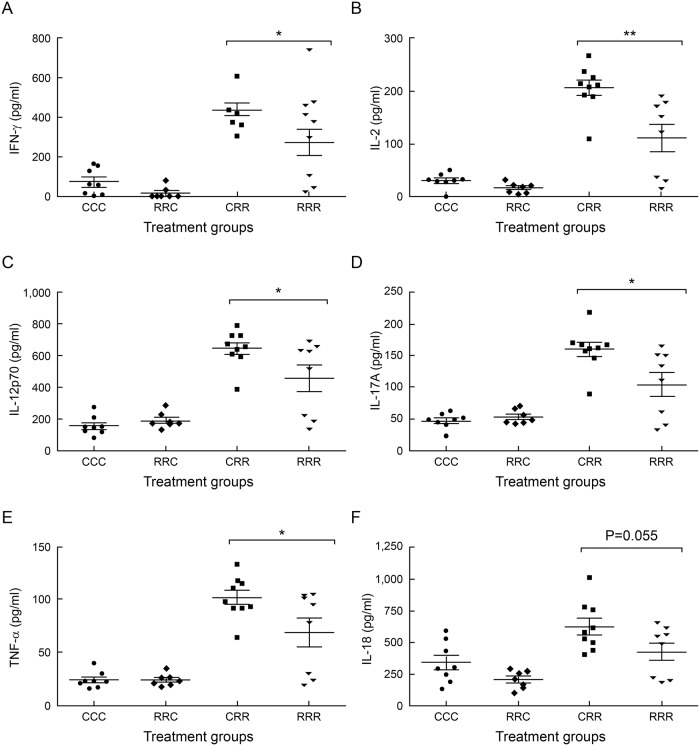
Maternal rrRSV infection is associated with altered immunity in offspring with recurrent postnatal rrRSV LRTI. Serum harvested from 28-day old weanling rats at the peak of rrRSV LRTI was analyzed using a multiplex cytokine/chemokine ELISA. Results demonstrate that maternal rrRSV infection reduces: (A) IFN-γ, (B) IL-2, (C) IL-12p70, (D) IL-17A, (E) TNF-α, and (F) IL-18 in offspring with recurrent early-life rrRSV LRTI (RRR) compared to controls from mothers not exposed to RSV (CRR). Data for 3 experiments were combined and expressed as the mean ± SEM of ≥7 rats per group. Two-tailed student’s *t* test: *P<0.05, **P<0.01 compared to RRR group.

Using serum obtained at D28 from sequentially rrRSV-inoculated weanlings, we utilized multiplex ELISA to assay levels of the same cytokines/chemokines array. We found that after repetitive postnatal rrRSV infection, weanlings (RRR) from RSV-infected mothers still had reduced levels of specific cytokines involved in innate and adaptive immunity to viral infections compared to CRR weanlings, including: IFN-γ (270.4 ± 66.94 pg/ml vs. 436.9 ± 30.57 pg/ml, *P*˂0.05; Fig 5A), IL-2 (111.9 ± 25.82 pg/ml vs. 207 ± 14.47 pg/ml, *P*<0.01; Fig 5B), IL-12p70 (460 ± 84.3 pg/ml vs. 650.50 ± 38.58 pg/ml, *P*<0.05; Fig 5C), IL-17A (104.2 ± 18.88 pg/ml vs. 159.9 ± 11.15 pg/ml, *P*<0.02; Fig 5D), and TNF-α (68.85 ± 13.61 pg/ml vs. 101.7 ± 6.66 pg/ml, *P*<0.04; Fig 5E). Importantly, with the possible exception of IL-18 (429 ± 70.13 pg/ml vs. 627.4 ± 65.29 pg/ml, *P* = 0.055; Fig 5F), which showed a strong but statistically non-significant trend, the same cytokines found to be suppressed in RR pups at D15 remained significantly reduced in RRR weanlings during recurrent early-life rrRSV LRTI at D28.

Systemic IL-12p70, IFN-γ, and TNF-α were significantly suppressed in RRR compared to CRR weanlings at D28, whereas lower airway CD4^+^ T and CD8^+^ T cell levels were shown to be equivalent. These findings support the concept that innate cytokine suppression in RRR weanlings results from impaired T_eff_ cell activation and not reduced infiltration of T_eff_ cells into the lower airways during recurrent early-life rrRSV LRTI. This hypothesis is also consistent with clinical reports of reduced CD8^+^ T cell activation, proliferation, and function during RSV LRTI leading to suppressed Th1 immunity hallmarked by deficient viral clearance, prolonged disease, and increased severity [30–32]. Additionally, CD3^+^ T cell counts in the lower airway of RRR weanlings were shown to be approximately 4.5-fold higher during recurrent postnatal rrRSV LRTI compared to CRR weanlings. Taken together, these data indicate maternal rrRSV infection conveys lasting effects on postnatal immune function possibly through the suppression of specific T-cell subsets during rrRSV reinfections.

In pups first inoculated with rrRSV on D10—but not again on D23—all cytokines and chemokines analyzed via multiplex ELISA were not significantly different across treatment groups at D28. While IL-2 and IFN-γ continued to display non-equivalent levels in vertically infected weanlings compared to controls, these differences were not significant (S1 Fig). Of particular importance for the proposed link between RSV and asthma, IL-5 was markedly reduced in RRR compared to CRR weanlings during recurrent postnatal rrRSV LRTI (360.6 ± 55.77 pg/ml vs. 488.1 ± 17.92 pg/ml; *P*˂0.05). We also found that IL-4 (90.46 ± 23.62 pg/ml vs. 145.7 ± 16 pg/ml; *P* = 0.0671) and IL-13 (84.3 ± 23.84 pg/ml vs. 138.7 ± 13.42 pg/ml; *P* = 0.0585) were lower in RRR compared to CRR weanlings, but these differences were not significant. Therefore, the differences in cytokine and chemokine expression observed between CRR and RRR weanlings at D28 seemingly diminish in the absence of secondary early-life rrRSV infection.

### Prenatal exposure to rrRSV is associated with increased postnatal NGF expression

**Fig 6:**
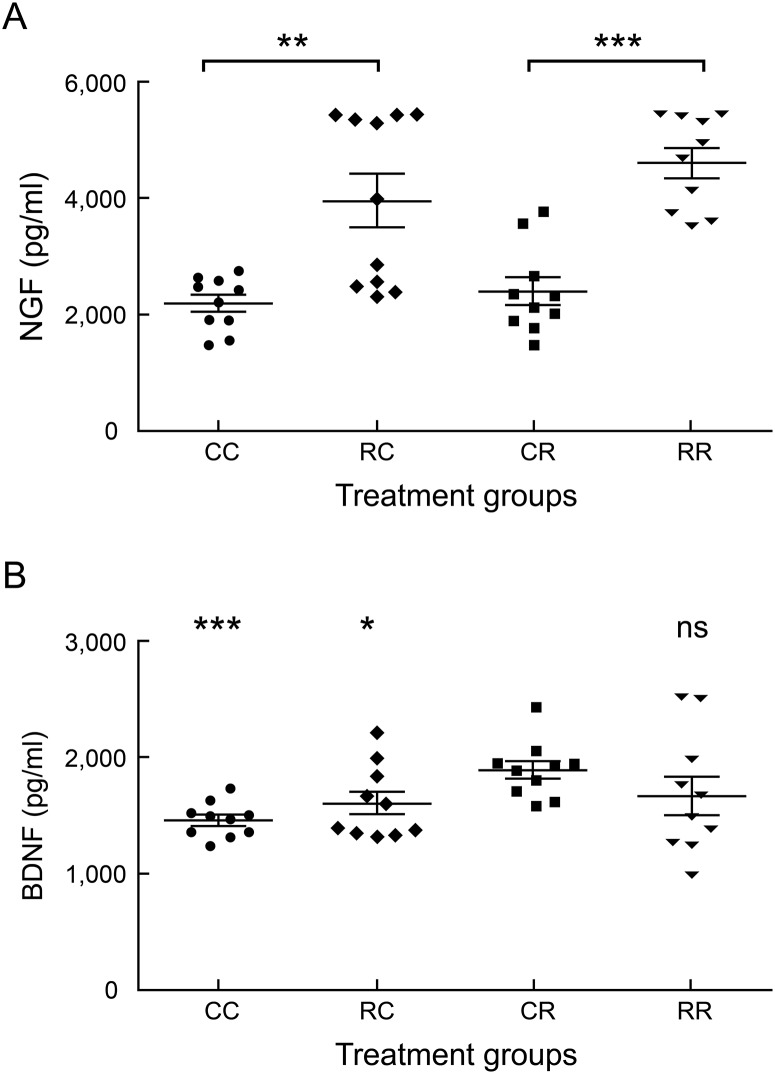
Prenatal rrRSV exposure is associated with increased postnatal NGF expression. (A) Maternal rrRSV infection significantly increased NGF expression in offspring compared to RSV-naïve controls both in the presence (RR vs. CR) and absence (RC vs. CC) of postnatal rrRSV LRTI. (B) BDNF levels did not increase because of prenatal rrRSV exposure, regardless of postnatal rrRSV LRTI status (CC vs. RC or RR), whereas postnatal rrRSV inoculation was associated with significantly increased BDNF in the absence of prenatal rrRSV exposure (CR vs. CC). Data for 3 experiments were combined and expressed as the mean ± SEM of 10 rats per group. Two-tailed student’s *t* test: *P<0.05, **P<0.01, ***P<0.001 as shown in Fig 6A or compared to the CR group in Fig 6B.

As we have previously shown elevated neurotrophin expression in vertically infected fetal rats [15], we sought to investigate what influence maternal rrRSV infection may have on neurotrophin expression in offspring with primary early-life rrRSV LRTI. Thus, we measured NGF and BDNF concentration in serum collected from pups at D15. We found higher NGF concentration in RR compared to CR pups at D15 (4,599 ± 254.9 pg/ml vs. 2,395 ± 236.9 pg/ml; *P*<0.0001; Fig 6A). These data suggest a pathogenic link between elevated NGF and increased response to nerve stimulation previously observed in vertically infected pups during early-life rrRSV LRTI [15].

In contrast, CR pups exhibited elevated BDNF (1,884 ± 76.97 vs. 1,455 ± 46.9 pg/ml; *P*<0.001) compared to non-infected CC pups at D15 (Fig 6B), but pups from infected mothers failed to display significantly elevated serum BDNF compared to non-infected CC controls at D15 either in the absence (RC; 1,601 ± 99.59 pg/ml; *P* = 0.2) or presence (RR; 1,659 ± 163.9 pg/ml; *P* = 0.245) of postnatal rrRSV inoculation. Moreover, while CR pups did exhibit significantly elevated serum BDNF levels compared to RC pups (1,884 ± 76.97 pg/ml vs. 1,601 ± 99.59 pg/ml; *P* = 0.037), no significant difference was observed between CR and RR pups at D15 (1,884 ± 76.97 pg/ml vs. 1,659 ± 163.9 pg/ml; *P* = 0.231), suggesting BDNF expression is not influenced by prenatal exposure to rrRSV.

### Prenatal rrRSV exposure alters lower airway smooth muscle contractility

**Fig 7:**
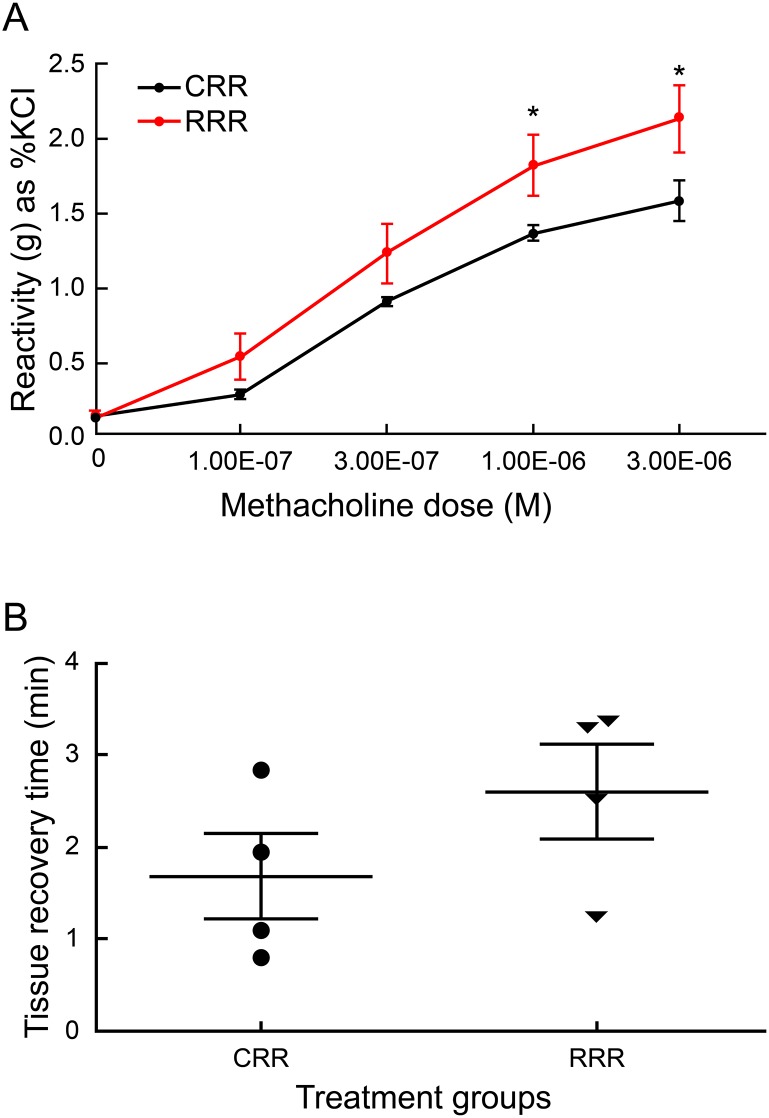
Prenatal rrRSV exposure is associated with hyperreactivity and prolonged contraction of lower airway smooth muscle. (A) Lower airway smooth muscle contraction in response to methacholine was measured *ex vivo* using tracheal segments harvested from 28-day old weanling rats during the peak of rrRSV LRTI. Baseline reactivity in response to KCl (80mM) was used to normalize data. (B) Tissue recovery time (TRT) required for the smooth muscle to return to baseline tone after a single 30-Hz pulse of electrical field stimulation was measured in minutes. Data for 3 experiments were combined and expressed as the mean ± SEM of 9 rats per group in Fig 7A, or 2 independent experiments combined and expressed as the mean ± SEM of 4 rats per group in Fig 7B. Two-tailed student’s *t* test: *P<0.05 compared to the CRR group.

Since we observed elevated serum NGF, altered lower airway immune cell patterns, and decreased anti-viral cytokine levels in RR pups at D15, we next sought to determine whether these effects were associated to changes in airway smooth muscle (ASM) reactivity and contractility after repetitive postnatal rrRSV infection. Tracheas were harvested on D28 and methacholine challenge was performed using a physiological muscle bath system. ASM reactivity was markedly elevated during recurrent postnatal rrRSV LRTI in RRR compared to CRR weanlings at methacholine concentrations of 1 × 10^−6^ M (1.81 ± 0.21 vs. 1.35 ± 0.6 gm as % KCl; *P*<0.05) and 3 × 10^−6^ M (2.12 ± 0.23 vs. 1.57 ± 0.14 gm as % KCl; *P*<0.05) respectively (Fig 7A). Additionally, the difference between RRR and CRR weanlings approached statistical significance at lower methacholine concentrations of 1 × 10^−7^ M (0.52 ± 0.16 vs. 0.27 ± 0.03 gm as % KCl; *P* = 0.065) and 3 × 10^−7^ M methacholine (1.22 ± 0.2 vs. 0.89 ± 0.03 gm as % KCl; *P* = 0.065).

We next sought to find additional endpoints to capture intrinsic ASM dysfunction. To do so, we identified and optimized a novel parameter which we termed “tissue recovery time” (TRT). To measure TRT, we performed *ex vivo* electrical field stimulation (EFS) on tracheal ring specimens using a 10-s pulse of electrical frequency at 30 Hz and recorded the length of time required for a specimen to reach its initial baseline resting tension. Our EFS single-frequency TRT data closely parallel and effectively support methacholine challenge findings (Fig 7B). Specifically, we observed a 35% increase of TRT in RRR compared to CRR weanlings (2.61 ± 0.5 min vs. 1.69 ± 0.46 min; *P* = 0.1130), which is equivalent to an average increase of 54.75 s in contraction time above resting baseline. This finding suggests that weanling rats from RSV-infected mothers require a greater amount of time (almost 1 full minute) to elapse before their lower airway smooth muscle returns to its resting state during secondary early-life rrRSV infection. Although the mechanism remains unclear, taken together our tissue bath data suggest that the lower airway smooth muscle of prenatally exposed rats is primed for both stronger and longer contractions in response to a triggering stimulus well after birth.

## Discussion

*In vivo* rodent models of vertical RSV transmission have shown that maternal-to-fetal transfer of this virus is possible and predisposes to airway hyperreactivity during a primary early-life infection with the same pathogen [15]. However, whether maternal RSV infection has consequences for the development of the offspring immune system and how it responds during a postnatal reinfection has not been characterized until now. Our results demonstrate that maternal RSV infection alters postnatal offspring immunity, resulting in lower airway lymphocyte profiles that are significantly different from that of a RSV-naïve host during a first postnatal RSV LRTI. Specifically, we found large CD3^+^ T cell elevations in the context of reduced CD4^+^ T, CD8^+^ T, and B cell populations, which remind of the findings reported by Welliver and colleagues in children succumbing to severe RSV disease [33]. One of the limitations of the present study, however, is lack of in depth analysis of RSV-specific T cell responses and immunologic memory development in our vertically infected rat pups, which we plan to examine in future studies.

As infants are infected with RSV during an early development window where host responses to the virus are incomplete and fail to prevent reinfection [1, 34–36], we studied whether maternal RSV infection affects lower airway immune cell counts during repeated postnatal RSV LRTI and observed sustained CD3^+^ T cell elevation in the lungs of weanling rats from infected mothers during a second early-life reinfection. Similar to that seen in our primary infection experiments, the CD3^+^ T cell population observed in weanlings from infected mothers consisted primarily of CD3^+^CD4^-^ and CD3^+^CD8^-^ T cells that were not present in controls from non-infected mothers. This finding suggests that maternal RSV infection facilitates the maintenance of phenotypically different CD3^+^ T cell subsets within the lower airways of offspring with recurrent early-life RSV LRTI, which might interfere with CD4^+^ T and CD8^+^ T cell activation and function. While remaining important to investigate, the influence of prenatal RSV exposure on lower airway T cell activation and gene expression profiles during postnatal RSV LRTI falls outside the scope of this study.

Another finding with potential clinical implications is that offspring from RSV-infected mothers displayed elevated B cell and monocyte lineage cell counts within their lower airways during repetitive postnatal RSV LRTI. Thus, the ineffective immunoglobulin production observed in several human studies [35–39] might arise from defective maturation of competent B cells and/or monocytes. Additionally, the combination of low cytokine levels and high B cell counts in RRR rats may be reflective of what has been reported in clinical studies by Mejias et al. [40] on whole blood gene expression, showing that specific T and B cell genes involved in both innate and adaptive immunity are consistently under-expressed in infants with the most severe RSV disease.

Our data also demonstrate that alterations of host anti-RSV immunity persist across consecutive early-life reinfections, suggesting that postnatal antiviral responses are programmed *in utero* through modification of the developing immune system. A possible explanation is that in our experiments the fetus is exposed to RSV in its preimmune phase, i.e., before the establishment of immunological self-tolerance is complete, and it is therefore conceivable that viral antigens might be recognized as self during an early-life reinfection. In support of this hypothesis, the prolonged IL-2 suppression in vertically infected rats during postnatal RSV LRTI suggests that those pups may be failing to recognize the virus as pathogenic and non-self, since IL-2 is produced physiologically as a result of naïve Th0-to-Th1 phenotype switching [27].

Our data are also well aligned with clinical reports of reduced IFN-γ levels correlating with greater disease severity in infants with RSV LRTI who fail to mount competent T-cell mediated immune responses [41, 42]. Taken together, these observations suggest that vertical RSV transmission might predispose to impaired CD4^+^ and CD8^+^ T cell-mediated immunity during early-life RSV LRTI. It will be necessary to thoroughly examine and confirm this hypothesis with future studies in order to determine whether the reduced host immunity we are observing is due to functional tolerance mediated by a currently unknown CD3^+^ T cell subset capable of inhibiting CD4^+^ and CD8^+^ T cell activation, or it is rather a consequence of transferred maternal immunity.

We have shown previously that vertical RSV transmission leads to NGF overexpression in fetuses extracted from infected dams, as well as in the lungs of offspring [15]. Accordingly, pharmacological blockade of TrkA, the cognate receptor for NGF, reduced AHR in vertically infected pups during early-life RSV reinfection [15]. In the present study, we observed nearly 2-fold higher NGF levels in pups from RSV infected-mothers compared to controls non-infected *in utero*. The translational relevance of these data derives from clinical evidence that early-life RSV infection is linked to chronic airway dysfunction in humans [3–8, 43], which is mediated at least in part by NGF [14, 15, 44]. Furthermore, increased NGF/TrkA axis expression in tracheal aspirates from mechanically ventilated infants with naturally occurring RSV LRTI suggests that disease severity closely parallels local concentrations of this neurotrophin [14]. As a potential practical application of these studies, NGF has been proposed as an effective biomarker to predict infant RSV LRTI severity [45].

Lastly, we found that dysfunctional host immunity and elevated NGF levels are associated with stronger and prolonged ASM contraction in vertically infected rats. Prenatal RSV exposure resulted in postnatal AHR in response to both *ex vivo* methacholine challenge and EFS, suggesting that RSV interferes with the development of both pre- and post-synaptic neuronal function. Furthermore, ASM of weanling rats from RSV-infected mothers required an appreciably greater amount of time to return to its baseline tone following contraction. Notably, vertical RSV transmission conveyed a lasting influence on ASM function observed across multiple, temporally spaced early-life RSV LRTI. The significance of these findings stems again from the known epidemiologic association between early-life RSV LRTI and the development of recurrent wheezing and chronic asthma well after the acute phase of infection has resolved [15, 46]. It is also important to note that direct stimulation of lower airway smooth muscle from pups with recurrent postnatal RSV LRTI produced significantly lower reactivity to methacholine in the absence of *in utero* exposure. This is important as it implies that prenatal RSV exposure is required for the development of postnatal AHR, whereas being infected one or more times in early-life may not be sufficient.

In conclusion, this study provides evidence that rats born to RSV infected mothers have altered immune responses, amplified neurotrophic signaling, and increased contractility of their lower airways smooth muscle during postnatal reinfections with the same virus. The most significant effects measured after primary early life infection were a sharp increase in CD3^+^CD4^neg^CD8^neg^ T cells combined with virtual suppression of the production of key Th1-type cytokines like IFN-γ and IL-2; a large increase in the expression of the prototypical neurotrophin NGF; and increased pre- and post-synaptic reactivity of the ASM combined with intrinsic hyper-contractility delaying its return to resting tone. These changes persisted—albeit to a lesser degree—after secondary reinfection and might provide a plausible explanation to the development of chronic airway dysfunction in a subpopulation of children with history of severe RSV infections in infancy.

## Materials and Methods

### Ethics statement

All procedures utilized in this study adhered to the NIH Guide for the Care and Use of Laboratory Animals and were reviewed and approved by the Institutional Animal Care and Use Committee (IACUC) of the Lerner Research Institute at Cleveland Clinic.

### Animals

Ten week old, pathogen-free Fischer 344 (F-344) rats (Envigo RMS, Indianapolis, IN) were housed under sterile conditions within a rat ABSL-2 facility using polycarbonate isolation cages mounted on racks providing positive individual ventilation with class-100 air at a rate of one cage change/minute (Lab Products, Seaford, DE). All cages and components including bedding, water hydra-packs, and chow were autoclaved before use and handled inside class-100 laminar flow hoods. Dams were pregnancy timed and were dosed with virus-free medium or inoculated with rrRSV at E12, as previously described [47].

### rrRSV preparation

Recombinant RSV-A_2_ expressing the red fluorescent protein (RFP) gene (rrRSV) was kindly provided by Dr. Mark Peeples (Nationwide Children’s Hospital, Columbus, OH) and Dr. Peter Collins (National Institutes of Health, Bethesda, MD) [48]. Expression of viable RFP requires successful full-length RSV replication and the rrRSV strain construct (BN1) used in these experiments is described elsewhere [48, 49]. Stock rrRSV was propagated using HEp-2 cells (ATCC CCL-23; American Type Culture Collection, Manassas, VA) in 1X DMEM with 10% fetal bovine serum. HEp-2 cells at 70% confluence were inoculated, harvested and titrated as described previously [15]. To obtain virus-free inoculum, HEp-2 cells were identically cultured and harvested.

### rrRSV inoculation

Dams received rrRSV suspension (4.0 × 10^6^ PFU) or an equal volume of virus-free medium by intratracheal (i.t.) instillation at E12 [15], and their litters were delivered at full term. At D10, pups received rrRSV (4.0 × 10^5^ PFU i.t.) or an equal volume of virus-free medium. This established 4 groups—CC, CR, RC, and RR—where ‘C’ stands for control and ‘R’ stands for rrRSV, with the left letter referring to the prenatal inoculation status and the right letter referring to the postnatal inoculation status. Terminal experiments were conducted 5 days post-inoculation (D15). For postnatal reinfection at D23, weanlings previously inoculated at D10 received another dose of rrRSV (5.0 × 10^5^ PFU) or an equal volume of virus-free medium. This established a distinct set of 4 additional groups—CCC, CRR, RCC, and RRR—with terminal experiments performed 5 days later (D28).

### Bronchoalveolar lavage

Rats were intubated using an 18-gauge blunted cannula inserted below the cricoid cartilage before 1.5 ml of chilled lavage buffer (1X PBS, 0.5% BSA, 0.5 mM EDTA, 25 mM HEPES) was squirted and retrieved. This procedure was repeated for a total of 2 independent lavages. Cells were stained for viability with Live/Dead Stain (Life Technologies, Carlsbad, CA), and then stained using FITC anti-CD45. Next, cells were stained with the following cocktail of secondary conjugated anti-rat monoclonal antibodies diluted per manufacturers’ instructions: V450 anti-CD4, APC anti-CD3, PE-Cy5 anti-CD45RA, PE-Cy7 anti-CD11b/c, and PE anti-CD8α (all from BD Biosciences, San Jose, CA). The choice to use CD45RA as a marker for B cells is a unique advantage of working with a rat model where mature B cells can be easily distinguished from other leukocytes on the basis of their expression of CD45 isoforms. Flow cytometry was performed with FACSAria (BD Biosciences) and data were analyzed using FlowJo software (TreeStar, Ashland, OR).

### Serum cytokine/chemokine and neurotrophin analysis

Blood was collected by cardiac puncture with a 27-gauge needle and immediately transferred to Microtainer SST tubes (BD, Franklin Lakes, NJ). Serum isolated per manufacturer’s protocol was analyzed for cytokines/chemokines using a MILLIPLEX MAP Rat Cytokine/Chemokine 27-Plex kit (EMD-Millipore, Billerica, MA) and a MAGPIX system (Luminex, Austin, TX). This multiplex ELISA detects the following mediators: EGF, eotaxin, fractalkine, G-CSF, GM-CSF, GRO/KC, IFN-γ, IL-1α, IL-1β, IL-2, IL-4, IL-5, IL-6, IL-10, IL-12p70, IL-13, IL-17A, IL-18, IP-10, Leptin, LIX, MCP-1, MIP-1α, MIP-2, RANTES, TNF-α, and VEGF. Data were generated using xMAP Technology (Luminex). Serum neurotrophins were analyzed with the NGF Emax and BDNF Emax immunoassays (Promega, Madison, WI) and a FlexStation3 plate reader (Molecular Devices, Sunnyvale, CA).

### Airway reactivity measurement

Airway smooth muscle contraction in response to exogenous cholinergic stimulation with methacholine (post-synaptic response) was measured *ex vivo* 5 days post-inoculation. We also evaluated muscle contraction resulting from endogenous neurotransmitter release from airway nerve terminals (pre-synaptic response) using electrical field stimulation (EFS). Explanted tracheas were bathed in modified Krebs-Henseleit solution (118 mM NaCl, 4.7 mM KCl, 0.6 mM MgSO_4_*7 H_2_O, 25 mM NaHCO_3_, 1.2 mM KH_2_PO_4_, 5.6 mM glucose, 2.5 mM CaCl_2_) under continuous aeration (95%O_2_/5% CO_2_) at 37°C.

In both assays, three cartilage ring-wide tracheal segments were suspended between stainless steel supports in tissue baths (Radnoti, LLC; Monrovia, CA). Passive tension was set at 0.3 gm during a tissue equilibration phase of 1 h. Isometric force changes were recorded using PowerLab Systems software (ADInstruments, Colorado Springs, CO). After reference contraction in response to KCl (80 mM), buffer was replaced and samples returned to resting tension.

To obtain cumulative dose-response curves, increasing concentrations of methacholine (Sigma-Aldrich, St. Louis, MO) were added to the bath solution and contraction force expressed in grams was normalized to KCl. EFS frequency-response curves were generated by increasing frequency incrementally from 0.1 to 30 Hz, using a submaximal voltage of 32 V, 10-ms pulse duration, and 11-s square wave trains. We also developed a strategy to quantify intrinsic muscle contractility by measuring the time required for specimens to return to resting tension after peak contraction during the 30-Hz EFS pulse interval (tissue recovery time, TRT).

### Statistics

All *in vivo* data are expressed as mean ± SEM. Two-tailed, unpaired Student’s *t*-test analysis was performed to determine statistical significance using Prism software (GraphPad, La Jolla, CA). P-value of ˂0.05 was considered statistically significant.

## Supporting Information

S1 Fig Cytokine levels normalize in the absence of recurrent rrRSV LRTI. Serum harvested from 28-day old weanling rats infected with rrRSV at D10, but not at D23. The concentrations of all cytokines and chemokines measured by multiplex ELISA in weanlings from RSV-infected mothers were not statistically different from control. Data are representative of 3 independent experiments, n ≥16 rats per group.
(TIF)

## References

[1] Wright M, Piedimonte G. Respiratory syncytial virus prevention and therapy: past, present, and future. Pediatr Pulmonol. 2011;46:324–47. 10.1002/ppul.21377 21438168

[2] Byington CL, Wilkes J, Korgenski K, Sheng X. Respiratory syncytial virus-associated mortality in hospitalized infants and young children. Pediatrics. 2015;135:e24–31. 10.1542/peds.2014-2151 25489019 PMC4279071

[3] Sims DG, Downham MA, Gardner PS, Webb JK, Weightman D. Study of 8-year-old children with a history of respiratory syncytial virus bronchiolitis in infancy. Br Med J. 1978;1:11–4. 620129 10.1136/bmj.1.6104.11 PMC1602461

[4] Mok JY, Simpson H. Outcome for acute bronchitis, bronchiolitis, and pneumonia in infancy. Arch Dis Child. 1984;59:306–9. 6721555 10.1136/adc.59.4.306 PMC1628672

[5] Stein RT, Sherrill D, Morgan WJ, Holberg CJ, Halonen M, Taussig LM, et al Respiratory syncytial virus in early life and risk of wheeze and allergy by age 13 years. Lancet. 1999;354:541–5. 10.1016/S0140-6736(98)10321-5 10470697

[6] Sigurs N, Bjarnason R, Sigurbergsson F, Kjellman B. Respiratory syncytial virus bronchiolitis in infancy is an important risk factor for asthma and allergy at age 7. Am J Respir Crit Care Med. 2000;161:1501–7. 10.1164/ajrccm.161.5.9906076 10806145

[7] Noble V, Murray M, Webb MS, Alexander J, Swarbrick AS, Milner AD. Respiratory status and allergy nine to 10 years after acute bronchiolitis. Arch Dis Child. 1997;76:315–9. 9166022 10.1136/adc.76.4.315 PMC1717138

[8] Openshaw PJM, Dean GS, Culley FJ. Links between respiratory syncytial virus bronchiolitis and childhood asthma: clinical and research approaches. Pediatr Infect Dis J. 2003;22:S58–S65. 10.1097/01.inf.0000053887.26571.eb 12671454

[9] Kim HW, Canchola JG, Brandt CD, Pyles G, Chanock RM, Jensen K, et al Respiratory syncytial virus disease in infants despite prior administration of antigenic inactivated vaccine. Am J Epidemiol. 1969;89:422–34. 4305198 10.1093/oxfordjournals.aje.a120955

[10] Chin J, Magoffin RL, Shearer LA, Schieble JH, Lennette EH. Field evaluation of a respiratory syncytial virus vaccine and a trivalent parainfluenza virus vaccine in a pediatric population. Am J Epidemiol. 1969;89:449–63. 4305200 10.1093/oxfordjournals.aje.a120957

[11] Piedimonte G, Perez MK. Respiratory syncytial virus infection and bronchiolitis. Pediatr Rev. 2014;35:519–30. 10.1542/pir.35-12-519 25452661 PMC5029757

[12] Lindsay RM, Harmar AJ. Nerve growth factor regulates expression of neuropeptide genes in adult sensory neurons. Nature. 1989;337:362–4. 10.1038/337362a0 2911387

[13] Bonini S, Lambiase A, Bonini S, Angelucci F, Magrini L, Manni L, et al Circulating nerve growth factor levels are increased in humans with allergic diseases and asthma. Proc Natl Acad Sci U S A. 1996;93:10955–60. 8855290 10.1073/pnas.93.20.10955 PMC38265

[14] Tortorolo L, Langer A, Polidori G, Vento G, Stampachiacchere B, Aloe L, et al Neurotrophin overexpression in lower airways of infants with respiratory syncytial virus infection. Am J Respir Crit Care Med. 2005;172:233–7. 10.1164/rccm.200412-1693OC 15879412

[15] Piedimonte G, Walton C, Samsell L. Vertical transmission of respiratory syncytial virus modulates pre- and postnatal innervation and reactivity of rat airways. PLoS One. 2013;8:e61309 10.1371/journal.pone.0061309 23637810 PMC3630224

[16] Manti S, Brown P, Perez MK, Piedimonte G. The role of neurotrophins in inflammation and allergy In: Litwack G, editor. Neurotrophins. Vitamins and Hormones, volume 104 Cambridge, Massachusetts: Academic Press/Elsevier; 2016. 10.1016/bs.vh.2016.10.010 28215300

[17] Baker CA, Swainson L, Lin DL, Wong S, Hartigan-O'Connor DJ, Lifson JD, et al Exposure to SIV in utero results in reduced viral loads and altered responsiveness to postnatal challenge. Sci Transl Med. 2015;7:300ra125 10.1126/scitranslmed.aac5547 26268312 PMC5100009

[18] Rohwedder A, Keminer O, Forster J, Schneider K, Schneider E, Werchau H. Detection of respiratory syncytial virus RNA in blood of neonates by polymerase chain reaction. J Med Virol. 1998;54:320–7. 9557299 10.1002/(sici)1096-9071(199804)54:4<320::aid-jmv13>3.0.co;2-j

[19] Graham BS, Johnson TR, Peebles RS. Immune-mediated disease pathogenesis in respiratory syncytial virus infection. Immunopharmacology. 2000;48:237–47. 10960663 10.1016/s0162-3109(00)00233-2

[20] Yui I, Hoshi A, Shigeta Y, Takami T, Nakayama T. Detection of human respiratory syncytial virus sequences in peripheral blood mononuclear cells. J Med Virol. 2003;70:481–9. 10.1002/jmv.10421 12767015

[21] Sweetman LL, Ng YT, Butler IJ, Bodensteiner JB. Neurologic complications associated with respiratory syncytial virus. Pediatr Neurol. 2005;32:307–10. 10.1016/j.pediatrneurol.2005.01.010 15866430

[22] Li XQ, Fu ZF, Alvarez R, Henderson C, Tripp RA. Respiratory syncytial virus (RSV) infects neuronal cells and processes that innervate the lung by a process involving RSV G protein. J Virol. 2006;80:537–40. 10.1128/JVI.80.1.537-540.2006 16352577 PMC1317531

[23] Torres JP, Gomez AM, Khokhar S, Bhoj VG, Tagliabue C, Chang ML, et al Respiratory syncytial virus (RSV) RNA loads in peripheral blood correlates with disease severity in mice. Respir Res. 2010;11:125 10.1186/1465-9921-11-125 20843364 PMC2946301

[24] Menchise A. Myocarditis in the setting of RSV bronchiolitis. Fetal Pediatr Pathol. 2011;30:64–8. 10.3109/15513815.2010.505632 21204669

[25] Kirin BK, Topic RZ, Dodig S. Hepatitis during respiratory syncytial virus infection—a case report. Biochem Med. 2013;23:112–6. 10.11613/BM.2013.014 PMC3900097 23457772

[26] Kim L. Respiratory Syncytial Virus (RSV) and RSV Vaccines 2016. https://www.cdc.gov/vaccines/acip/meetings/downloads/slides-2016-06/rsv-02-kim.pdf.

[27] Wu J, Xie A, Chen W. Cytokine regulation of immune tolerance. Burns Trauma. 2014;2:11–7. 27574641 10.4103/2321-3868.124771 PMC4994505

[28] Jozwik A, Habibi MS, Paras A, Zhu J, Guvenel A, Dhariwal J, et al RSV-specific airway resident memory CD8+ T cells and differential disease severity after experimental human infection. Nat Commun. 2015;6:10224 10.1038/ncomms10224 26687547 PMC4703893

[29] Darwich L, Coma G, Pena R, Bellido R, Blanco EJ, Este JA, et al Secretion of interferon-gamma by human macrophages demonstrated at the single-cell level after costimulation with interleukin (IL)-12 plus IL-18. Immunology. 2009;126:386–93. 10.1111/j.1365-2567.2008.02905.x 18759749 PMC2669819

[30] Gonzalez PA, Carreno LJ, Bueno SM, Riedel CA, Kalergis AM. Understanding respiratory syncytial virus infection to improve treatment and immunity. Curr Mol Med. 2013;13:1122–39. 23157678 10.2174/1566524011313070007

[31] Gonzalez PA, Bueno SM, Riedel CA, Kalergis AM. Impairment of T cell immunity by the respiratory syncytial virus: targeting virulence mechanisms for therapy and prophylaxis. Curr Med Chem. 2009;16:4609–25. 19903147 10.2174/092986709789760724

[32] Munir S, Hillyer P, Le Nouen C, Buchholz UJ, Rabin RL, Collins PL, et al Respiratory syncytial virus interferon antagonist NS1 protein suppresses and skews the human T lymphocyte response. PLoS Pathog. 2011;7:e1001336 10.1371/journal.ppat.1001336 21533073 PMC3080852

[33] Welliver TP, Garofalo RP, Hosakote Y, Hintz KH, Avendano L, Sanchez K, et al Severe human lower respiratory tract illness caused by respiratory syncytial virus and influenza virus is characterized by the absence of pulmonary cytotoxic lymphocyte responses. J Infect Dis. 2007;195:1126–36. 10.1086/512615 17357048 PMC7109876

[34] Shay DK, Holman RC, Newman RD, Liu LL, Stout JW, Anderson LJ. Bronchiolitis-associated hospitalizations among US children, 1980–1996. JAMA. 1999;282:1440–6. 10535434 10.1001/jama.282.15.1440

[35] Glezen WP, Taber LH, Frank AL, Kasel JA. Risk of primary infection and reinfection with respiratory syncytial virus. Am J Dis Child. 1986;140:543–6. 3706232 10.1001/archpedi.1986.02140200053026

[36] Hall CB, Walsh EE, Long CE, Schnabel KC. Immunity to and frequency of reinfection with respiratory syncytial virus. J Infect Dis. 1991;163:693–8. 2010624 10.1093/infdis/163.4.693

[37] Habibi MS, Jozwik A, Makris S, Dunning J, Paras A, DeVincenzo JP, et al Impaired antibody-mediated protection and defective IgA B-cell memory in experimental infection of adults with respiratory syncytial virus. Am J Respir Crit Care Med. 2015;191:1040–9. 10.1164/rccm.201412-2256OC 25730467 PMC4435460

[38] Henderson FW, Collier AM, Clyde WA Jr., Denny FW. Respiratory syncytial virus infections, reinfections and immunity. A prospective, longitudinal study in young children. N Engl J Med. 1979;300:530–4. 10.1056/NEJM197903083001004 763253

[39] Hall CB, Long CE, Schnabel KC. Respiratory syncytial virus infections in previously healthy working adults. Clin Infect Dis. 2001;33:792–6. 10.1086/322657 11512084

[40] Mejias A, Dimo B, Suarez NM, Garcia C, Suarez-Arrabal MC, Jartti T, et al Whole blood gene expression profiles to assess pathogenesis and disease severity in infants with respiratory syncytial virus infection. PLoS Med. 2013;10:e1001549 10.1371/journal.pmed.1001549 24265599 PMC3825655

[41] Bont L, Heijnen CJ, Kavelaars A, van Aalderen WM, Brus F, Draaisma JM, et al Local interferon-gamma levels during respiratory syncytial virus lower respiratory tract infection are associated with disease severity. J Infect Dis. 2001;184:355–8. 10.1086/322035 11443563

[42] Mella C, Suarez-Arrabal MC, Lopez S, Stephens J, Fernandez S, Hall MW, et al Innate immune dysfunction is associated with enhanced disease severity in infants with severe respiratory syncytial virus bronchiolitis. J Infect Dis. 2013;207:564–73. 10.1093/infdis/jis721 23204162 PMC3611762

[43] Murray M, Webb MS, O'Callaghan C, Swarbrick AS, Milner AD. Respiratory status and allergy after bronchiolitis. Arch Dis Child. 1992;67:482–7. 1580676 10.1136/adc.67.4.482 PMC1793348

[44] Hu C, Wedde-Beer K, Auais A, Rodriguez MM, Piedimonte G. Nerve growth factor and nerve growth factor receptors in respiratory syncytial virus-infected lungs. Am J Physiol Lung Cell Mol Physiol. 2002;283:L494–502. 10.1152/ajplung.00414.2001 12114213

[45] Brown PM, Schneeberger DL, Piedimonte G. Biomarkers of respiratory syncytial virus (RSV) infection: specific neutrophil and cytokine levels provide increased accuracy in predicting disease severity. Paediatr Respir Rev. 2015;16:232–40. 10.1016/j.prrv.2015.05.005 26074450 PMC4656140

[46] Piedimonte G, Hegele RG, Auais A. Persistent airway inflammation after resolution of respiratory syncytial virus infection in rats. Pediatr Res. 2004;55:657–65. 10.1203/01.PDR.0000112244.72924.26 14711892

[47] Sasser JM, Baylis C. The natriuretic and diuretic response to dopamine is maintained during rat pregnancy. Am J Physiol Renal Physiol. 2008;294:F1342–4. 10.1152/ajprenal.00067.2008 18400873 PMC4356245

[48] Guerrero-Plata A, Casola A, Suarez G, Yu X, Spetch L, Peeples ME, et al Differential response of dendritic cells to human metapneumovirus and respiratory syncytial virus. Am J Respir Cell Mol Biol. 2006;34:320–9. 10.1165/rcmb.2005-0287OC 16284360 PMC2644197

[49] Techaarpornkul S, Barretto N, Peeples ME. Functional analysis of recombinant respiratory syncytial virus deletion mutants lacking the small hydrophobic and/or attachment glycoprotein gene. J Virol. 2001;75:6825–34. 10.1128/JVI.75.15.6825-6834.2001 11435561 PMC114409

