# Supplementary figures and images for: Prenatal Exposure to Respiratory Syncytial Virus Alters Postnatal Immunity and Airway Smooth Muscle Contractility during Early-Life Reinfections
Source: PLoS One. 2017 Feb 8;12(2):e0168786. doi: 10.1371/journal.pone.0168786 (PMC5298216; doi:10.1371/journal.pone.0168786)

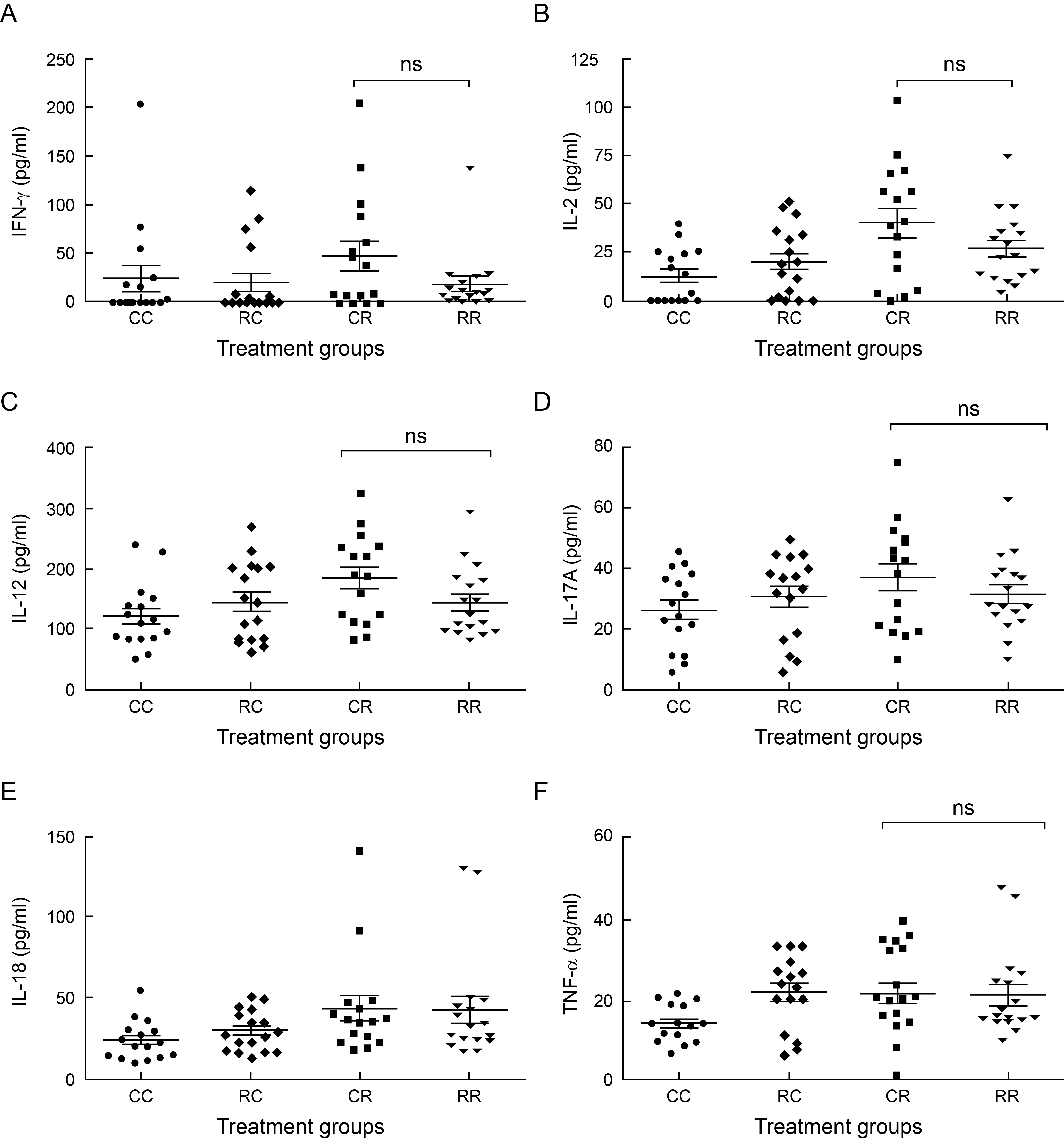

Supplement: S1 Fig — Serum harvested from 28-day old weanling rats infected with rrRSV at D10, but not at D23. The concentrations of all cytokines and chemokines measured by multiplex ELISA in weanlings from RSV-infected mothers were not statistically different from control. Data are representative of 3 independent experiments, n ≥16 rats per group. (TIF) [file pone.0168786.s001.tif]
